# Incidence of the *CHEK2* Germline Mutation and Its Impact on Clinicopathological Features, Treatment Responses, and Disease Course in Patients with Papillary Thyroid Carcinoma

**DOI:** 10.3390/cancers13030470

**Published:** 2021-01-26

**Authors:** Danuta Gąsior-Perczak, Artur Kowalik, Krzysztof Gruszczyński, Agnieszka Walczyk, Monika Siołek, Iwona Pałyga, Sławomir Trepka, Estera Mikina, Tomasz Trybek, Janusz Kopczyński, Agnieszka Suligowska, Rafał Ślusarczyk, Agnieszka Gonet, Jarosław Jaskulski, Paweł Orłowski, Magdalena Chrapek, Stanisław Góźdź, Aldona Kowalska

**Affiliations:** 1 Collegium Medicum, Jan Kochanowski University, 25-317 Kielce, Poland; a.walczyk@post.pl (A.W.); iwonapa@tlen.pl (I.P.); slavello@wp.pl (S.T.); r.slusarczyk@poczta.onet.pl (R.Ś.); agonet@poczta.onet.pl (A.G.); jaroslawja@poczta.fm (J.J.); pawelor@interia.pl (P.O.); stanislawgo@onkol.kielce.pl (S.G.); aldonako@onkol.kielce.pl (A.K.); 2 Endocrinology Clinic, Holycross Cancer Center, Artwińskiego 3, 25-734 Kielce, Poland; esterami@tlen.pl (E.M.); trytom1@tlen.pl (T.T.); a.suligowska@wp.pl (A.S.); 3 Department of Molecular Diagnostics, Holycross Cancer Center, Artwińskiego 3, 25-734 Kielce, Poland; arturko@onkol.kielce.pl (A.K.); gruszczynski.k@wp.pl (K.G.); 4 Division of Medical Biology, Institute of Biology Jan Kochanowski University, Uniwersytecka 7, 25-406 Kielce, Poland; 5 Genetic Clinic, Holycross Cancer Center, 25-734 Kielce, Poland; monika.siolek@wp.pl; 6 Department of Surgical Oncology, Holycross Cancer Center, Artwińskiego 3, 25-734 Kielce, Poland; 7 Surgical Pathology, Holycross Cancer Center, Artwińskiego 3, 25-734 Kielce, Poland; januszko@onkol.kielce.pl; 8 Faculty of Natural Sciences, Jan Kochanowski University, 25-406 Kielce, Poland; magdalena.chrapek@ujk.edu.pl; 9 Clinical Oncology, Holycross Cancer Center, Artwińskiego 3, 25-734 Kielce, Poland

**Keywords:** papillary thyroid cancer, *CHEK2*, *CHEK2* missense mutation, *CHEK2* truncating mutation, risk stratification, IVS2 + 1G >A, del5395, 1100delC, I157T

## Abstract

**Simple Summary:**

The aim of our study was to evaluate whether the *CHEK2* mutation was a predictor of poorer clinical course in patients with papillary thyroid cancer. The study included 1547 patients from a single center in Poland, in whom the presence and variant of the *CHEK2* mutation were determined. Two hundred and forty patients were found to carry this mutation. We found significant association of the *CHEK2* truncating variant with vascular invasion and intermediate or high initial risk of recurrence/persistence, whereas this relationship was not found in case of the missense *CHEK2* variant. Neither the truncating nor the missense mutations were associated with worse primary treatment response and outcome of the disease.

**Abstract:**

The *CHEK2* gene is involved in the repair of damaged DNA. *CHEK2* germline mutations impair this repair mechanism, causing genomic instability and increasing the risk of various cancers, including papillary thyroid carcinoma (PTC). Here, we asked whether *CHEK2* germline mutations predict a worse clinical course for PTC. The study included 1547 unselected PTC patients (1358 women and 189 men) treated at a single center. The relationship between mutation status and clinicopathological characteristics, treatment responses, and disease outcome was assessed. *CHEK2* mutations were found in 240 (15.5%) of patients. A *CHEK2* I157T missense mutation was found in 12.3%, and *CHEK2* truncating mutations (IVS2 + 1G > A, del5395, 1100delC) were found in 2.8%. The truncating mutations were more common in women (*p* = 0.038), and were associated with vascular invasion (OR, 6.91; *p* < 0.0001) and intermediate or high initial risk (OR, 1.92; *p* = 0.0481) in multivariate analysis. No significant differences in these parameters were observed in patients with the I157T missense mutation. In conclusion, the *CHEK2* truncating mutations were associated with vascular invasion and with intermediate and high initial risk of recurrence/persistence. Neither the truncating nor the missense mutations were associated with worse primary treatment response and outcome of the disease.

## 1. Introduction

Thyroid cancer is the most common cancer of the endocrine glands, accounting for 1–2% of all malignant neoplasms [1]. Papillary thyroid cancer (PTC) accounts for the vast majority of all thyroid cancers and has a favorable prognosis [2]. In recent years, increasing use of sensitive diagnostic procedures has led to a very rapid increase in the detection of differentiated thyroid cancer (mainly PTC with a low degree of malignancy) worldwide, including in Poland [3,4,5,6]. The study of the Surveillance, Epidemiology, and End Results-9 (SEER-9) dataset showed a real increase in incidence of the advanced-stage PTC [7]. The increase in PTC incidence has been associated, among others, with environmental factors, such as radiation exposure, volcanic ash or chemical agents such as pesticides [8]. However, the increase in detection has not translated into a decrease in mortality rate, despite the fact that thyroid cancer is over-diagnosed and over-treated [3,9]. Indeed, 20–30% of PTC patients experience recurrence after 15–20 years of follow-up and have poor prognosis due to distant metastases, which are sometimes fatal [10]. However, the highest risk of recurrence is within the first 5 years of follow-up [11]. Adequate stratification is crucial if we are to discriminate patients who require aggressive treatment from the start from those who will have a benign course.

Several factors predict recurrence, including the histopathological characteristics of the tumor and *BRAFV600E* mutation status, which is now included in the latest system of risk stratification of structural recurrence proposed by the American Thyroid Association (ATA 2015) [12]. The aforementioned ATA guidelines state that *BRAF V600E* mutation status can help with postoperative risk stratification and should be considered only in the context of clinicopathological risk factors; the guidelines do not recommend routine *BRAF V600E* mutation analysis [12]. Recently, it was emphasized that an unfavorable disease course is associated with mutations in the promoter of the gene encoding telomerase reverse transcriptase (*TERT*), and with the coexistence of *BRAF V600E* and *TERT* promoter mutations [13,14,15,16,17]. Researchers around the world continue to search for ideal molecular markers not only in tumor tissue obtained after surgery, but also in peripheral blood samples obtained before surgery; such markers would be a breakthrough in PTC prognostication and would not only contribute to the detection of cancer at an early stage (when the chances of cure are very high), but would also help to identify a subgroup of aggressive PTCs and be a useful predictor of clinical course prior to thyroid surgery [18,19,20,21,22,23,24,25,26,27,28,29,30,31].

In recent years, polymorphisms and mutations in genes within the ATM-BRCA1-*CHEK2* DNA repair pathway have been investigated in various types of cancer, including thyroid cancer [22,23,32]. One of the key tumor-suppressor genes involved in cell cycle checkpoint control, DNA damage response signaling, and the regulation of apoptosis and cell aging is checkpoint kinase 2 (*CHEK2*) [33,34,35]. The serine-threonine kinase mutated ataxia-telangiectasia gene (ATM) is activated in response to double-stranded DNA damage; ATM then phosphorylates and activates *CHEK2*, and *CHEK2* phosphorylates downstream cell cycle regulators such as p53, Cdc25, and BRCA1. The ultimate outcome of this signal chain is arrest of the cell cycle at the G1 and G2 phases, before cells enter mitosis [35,36]. When the cell cycle stops, the DNA repair system is activated. If DNA damage is not repaired, apoptosis is activated to remove the damaged cell from the body. Mutations in genes encoding proteins involved in DNA repair processes affect their stability or activity, which may contribute to neoplastic changes in cells [37,38]. Loss of kinase function due to mutations in the *CHEK2* gene is associated with an increased risk of developing a variety of sporadic and hereditary malignancies, including PTC [32,37,39]. In the Polish population, there are four different mutation variants of the *CHEK2* gene: three truncating mutations (1100delC, IVS2 + 1G > A, and del5395) and one missense mutation (I157T) that results in an amino acid change in the *CHEK2* protein (isoleucine to threonine), which is associated with a moderately increased risk of sporadic PTC [22,23,32,39,40]. Mutations in the *CHEK2* gene occur in 0–15.6% of PTC patients [23,32,38,41,42,43]. According to the Cancer Genome Atlas (TCGA; 2014), disruption of the DNA repair mechanism may trigger the development of an aggressive form of PTC [42]. Here, we assessed whether *CHEK2* germline mutations are a useful predictor of PTC with an adverse clinical course. We analyzed 1547 patients, all diagnosed with PTC and treated at a single center in Poland, for the presence of *CHEK2* germline mutations to assess how common the events are, and whether a particular variant is associated with specific clinicopathological features, response to primary treatment, and disease outcome.

## 2. Materials and Methods

### 2.1. Patients and Study Design

The study group comprised 1547 unselected PTC patients from Central and Eastern Poland, all of whom were diagnosed and treated at a single center (Holycross Cancer Center in Kielce). Patients were enrolled during follow-up visits to the endocrinology outpatient department in 2011–2018. Next, they were routinely referred to the genetics outpatient department to assess the risk of familial cancer. All patients provided signed informed consent and a blood samples for DNA analysis. The control group comprised 468 age- and sex-matched cancer-free adults. All patients and controls were Caucasian. The study excluded patients who did not provide written consent to molecular testing or who were lost to follow-up due to reasons other than death. Demographic and clinicopathological data, including sex, age at diagnosis of PTC, tumor diameter, histological PTC variant, multifocality, nodal and distant metastases, extrathyroidal extension, vascular invasion, initial risk stratification, response to primary treatment, and disease outcome (persistence/recurrence/death) were available for all analyzed cases and were reviewed retrospectively. Age in the control group was reported when blood was sampled for *CHEK2* mutation analysis. Post-operative assessment of TNM was reclassified according to the latest (8th) edition of the American Joint Committee on Cancer (AJCC)/Union for International Cancer Control (UICC) TNM, and the modified ATA initial risk stratification system (low, intermediate, and high risk of recurrence/persistence) [12,44]. The pNx characteristic was reclassified clinically as N0b or N1, and the Mx characteristic was reclassified as M0 or M1, according to the 8th edition of the TNM American Joint Committee on Cancer (AJCC)/UICC classification system, as described previously [45]. The follow-up summary for the current study was dated 31 May 2020. All subjects provided informed consent before participating in the study. The study was conducted in accordance with the Helsinki Declaration and the protocol was approved by the Ethics Committee of the Świętokrzyska Medical Chamber (code of ethics: 16/200-VII and 58/2018).

### 2.2. Management and Follow-Up Protocols

All patients underwent surgery as the primary treatment. The scope of surgery and the operating procedures in our center have been described previously [46]. All patients with a stage higher than pT1aN0-xM0 were eligible for treatment with 131-I. Protocols for post-operative evaluation of laboratory and imaging data, 131-I treatment, and tests and procedures of assessing responses to primary treatment, were carried out in accordance with ATA (excellent/no evidence of disease/indeterminate, biochemically, or structurally incomplete disease) guidelines, and reclassified according to the system of delayed risk stratification according to ATA recommendations adopted in our center, as described previously [45,47,48]. After the surgery, patients were treated with levothyroxine. The degree of TSH suppression depended on the initial risk and treatment response assessment, in accordance with the ATA recommendations [12,49]. Risk stratification was repeated continuously, and response categories were updated during follow-up in accordance with ATA guidelines [12]. Recurrence was defined as biochemical or structural evidence of disease following a period of no evidence of disease (NED) for at least 12 months after initial therapy (surgery + I131). In patients not treated with 131-I, biochemical tests such as serum Tg and TgAb levels were not used as criteria for recurrent/persistent disease [12,50].

### 2.3. End of Follow-Up and Oncological Assessment (31 May 2020)

The clinical condition of the patients was assessed on the basis of the medical records. Patients were classified as NED, indeterminate response, persistent disease, cancer-related death, and death from other causes in accordance with the latest ATA guidelines [12].

### 2.4. Detection of CHEK2 Mutations

The Micro AX Blood Gravity Kit (A&A Biotechnology, Gdańsk, Poland) was used to isolate DNA from whole-blood samples (100 µL collected in EDTA tubes) within 12 h. DNA samples were eluted in 120 µL buffer E, and CHEK mutation genotyping was performed using TaqMan PCR (Life Technologies Corporation, Pleasanton, CA, USA) (I157T) or allele-specific PCR and chip electrophoresis (IVS2 + 1G > A del5395, and 1100delC). Detected mutations (I157T, IVS2 + 1G > A, and 1100delC) were confirmed by Sanger sequencing. The methods of DNA isolation and genotyping, as well as the algorithm for molecular diagnostic research, were described in detail in our previous work [43].

### 2.5. Statistical Analysis

Continuous data are presented as the mean (standard deviation) and median (lower/upper quartiles and ranges (minimum and maximum)). Categorical data are presented as numbers and percentages. Group comparisons were performed using the Chi-square or Fisher’s exact test (categorical variables), the *t*-test (continuous, normally distributed variables), or the Mann–Whitney test (continuous, non-normally distributed variables). Normality of distribution was checked using the Shapiro–Wilk test. Odds ratio (OR) and 95% confidence intervals (95% CI) were calculated using logistic regression models. Multivariate logistic regression models included variables that were statistically significant in univariate analysis. A two-tailed *p*-value < 0.05 was considered statistically significant. All statistical analyses were performed using the R software package (version 3.6.2).

## 3. Results

### Baseline Characteristics

The clinicopathological characteristics of PTC patients, the molecular status of the *CHEK2* mutations, the primary treatment response, and disease outcomes for all 1547 cases are presented in Table 1. Of the 1547 unselected Caucasian patients (median age, 50 years; range, 15–85), the majority were women (1358; 87.8%), and 990 (64.0%) were under 55 years of age. The mean (±standard deviation) tumor diameter was 11.6 ± 11.5 mm (range, 0.3–110 mm). Primary tumor stage was pT1a, identified in 980 (63.3%) cases, and the dominant classic variant of PTC was found in 1111 (71.8%) cases. Gross extrathyroidal extension was identified in 25 (1.6%) cases, vascular invasion in 72 (4.7%), histologically verified metastases to lymph nodes in 225 (14.5%), and distant metastases in 15 (1.0%). Ten patients (0.7%) had more advanced (stage III/IV) disease according to the updated 8th AJCC/tumor-node-metastasis (TNM) staging system, and 404 (26.1%) had multifocal disease. A mutation in the *CHEK2* gene was found in 240 (15.5%) patients. The dominant mutation was the I157T missense mutation, found in 189 (12.3%). Truncating mutations (IVS2 + 1G > A, del5395, or 1100delC) were found in 44 (2.8%). Coexistence of two mutations was found in 7 (0.5%) patients. No mutation in the *CHEK2* gene was identified in 1307 (84.5%) patients. According to the latest ATA guidelines [12], 427 patients (27.6%) were classified as intermediate risk (IR) and 65 (4.2%) as high risk (HR). Treatment with 131-I was given to 1054 (68.1%) patients (activity = 1100–3700 MBq depending on advancement stage according TNM classification). Of these, 176 (11.4%) patients received 131-I more than once. The remaining 493 (31.9%) patients had a single thyroid-restricted PTC focus of ≤1 cm in diameter, no nodal or distant pT1aN0-xM0 metastases, no vascular invasion, and no aggressive histological variant of PTC. They received no treatment with 131-I. A very good response to primary treatment was found across the entire study group (1259 (81.4%) patients). The median follow-up time was 6 years (range, 1–34). No cancer-related deaths have been reported to date. However, 16 (1.0%) patients died due to other causes. At the end of follow-up, 102 (6.6%) patients were indeterminate, 12 (0.8%) had biochemically persistent disease, and ten (0.6%) had structurally persistent disease. Recurrence after a period of no evidence of disease (NED) occurred in 19 patients (1.2%).

#### 3.1.1. Relationship between the Genotype of *CHEK2* Mutation and PTC Risk

A *CHEK2* mutation (any of the four types of mutation) was observed in 240/1547 (15.5%) of unselected PTC patients versus 28/468 (6.0%) of the age and sex-matched control group (odds ratio (OR), 2.89; 95% confidence interval (CI), 1.92–4.33; *p* < 0.0001) (Table 2). The median age of the study group was 50 years (range, 15–85) and that in the control group was 50 years (range, 14–76) (*p* = 0.8521). In the study group, women accounted for 1358/1547 (87.8%) of cases and men for 189/1547 (12.2%) of cases. In the control group, women accounted for 415/468 (88.7%) of cases and men for 53/468 (11.3%) of cases (*p* = 0.6028). Both truncating heterozygous mutations (IVS2 + IG > A, 1100delC, and del5395) and the *CHEK2* I157T missense heterozygous mutation were associated with an increased risk of PTC. Heterozygous truncating mutations (IVS2 + IG > A, 1100delC, and del5395) in *CHEK2* occurred in 44/1547 (2.8%) unselected patients and in 3/468 (0.6%) controls (OR, 4.54; 95% CI, 1.40–14.68; *p* = 0.0116). The *CHEK2* I157T missense mutation occurred in 189/1547 (12.3%) unselected patients and in 25/468 (5.3%) controls (OR, 2.47; 95% CI, 1.60–3.79; *p* < 0.0001). The *CHEK2* I157T missense heterozygous mutation occurred in 182/1547 (11.8%) unselected patients compared with 25/468 (5.3%) controls (OR, 2.36; 95% CI, 1.53–3.64; *p* < 0.0001). By contrast, the *CHEK2* I157T missense homozygous mutation occurred in 7/1547 (0.5%) unselected patients and in none of the controls (*p* = 0.3641). Coexistence of the missense I157T heterozygous mutation and truncating heterozygous (IVS2 + IG > A and del5395) *CHEK2* mutations was found in 5/1547 (0.3%) unselected patients and in none of the controls (*p* = 0.5961). Coexistence of the missense I157T heterozygous mutation and the truncating heterozygous IVS2 + IG > A *CHEK2* mutation was found in 3/1547 (0.2%) unselected patients and in none of the controls, and coexistence of the I157T missense heterozygous and the truncating del5395 heterozygous *CHEK2* mutation was found in 2/1547 (0.1%) unselected patients and in none of the controls (*p* = 1.0 and *p* = 1.0, respectively). Coexistence of the truncating heterozygous (IVS2 + IG > A) mutation and truncating heterozygous (del5395) mutation was found in 2/1547 (0.1%) unselected patients and in none of the controls (*p* = 1.0).

#### 3.1.2. Relationship between *CHEK2* Mutation Status and *CHEK2* Wild-Type (WT) in Terms of Clinicopathological Characteristics, Responses to Therapy, and Disease Outcomes

Table 3 shows the relationships between individual clinical/pathological characteristics, responses to treatment, and disease outcomes and *CHEK2* mutations in the 1547 unselected PTC patients. There was no significant relationship between patients with any *CHEK2* mutation and *CHEK2* WT in terms of sex, age at diagnosis, tumor size, histopathological variant of multifocality, LN metastases, distant metastases and extrathyroidal extension, vascular invasion, and more advanced clinical stage. There was no significant relationship between the groups in terms of intermediate or high risk of recurrence/persistence, 131-I treatment, a worse response to primary treatment (indeterminate, biochemically incomplete, structurally incomplete), and disease outcome (persistent disease/recurrence/death).

#### 3.1.3. Relationship between *CHEK2* I157T Missense Heterozygous Mutation and *CHEK2* WT with Respect to Clinicopathological Characteristics, Responses to Therapy, and Disease Outcomes

The relationship between individual clinicopathological characteristics, responses to treatment, and disease outcome for 182 patients with the I157T missense heterozygous variant of the *CHEK2* mutation and those with *CHEK2* WT is presented in Table 3. There were no significant relationships between the groups in terms of sex, age at diagnosis, tumor size, histopathological variant, multifocality, metastases to LN, distant metastases, extrathyroidal extension, vascular invasion, and more advanced clinical stage. There were no significant relationships between the groups with respect to intermediate and high risk of recurrence/persistence, 131-I treatment, a worse response to primary treatment (indeterminate, biochemically incomplete, structurally incomplete), and disease outcome (persistent disease/recurrence/death).

#### 3.1.4. Relationship between the Heterozygous Truncating *CHEK2* Mutation Variants (IVS2 + 1G > A, del5395, and 1100delC) and *CHEK2* WT with respect to Clinicopathological Characteristics, Responses to Therapy, and Disease Outcomes

The clinicopathological characteristics, responses to treatment, and disease outcomes of 44 patients with heterozygous truncating *CHEK2* mutation variants (IVS2 + 1G > A, del5395, or 1100delC) were compared with those harboring the *CHEK2* WT (Table 3). There was no significant relationship between the two groups in terms of age at diagnosis, multifocality, LN metastases, distant metastases, extrathyroidal extension, and more advanced clinical stage. Heterozygous truncating *CHEK2* mutation variants (IVS2 + 1G > A, del5395, and 1100delC) were more common than *CHEK2* WT (*p* = 0.038) in women. Differences in tumor size between the groups were statistically significant (*p* = 0.0356). Tumors >20–40 mm were more common in patients with a *CHEK2* heterozygous truncating mutation (IVS2 + 1G > A, del5395, or 1100delC) (22.7%) than in those with *CHEK2* WT (10.2%). Differences in histological variant of PTC were also statistically significant (*p* = 0.0397). The classic histological variant of PTC was more common in patients with *CHEK2* WT (71.6%) than in patients with a heterozygous *CHEK2* truncating mutation (IVS2 + 1G > A, del5395, or 1100delC) (61.4%). The follicular and oxyphilic variants were more common in patients with a heterozygous *CHEK2* truncating mutation (IVS2 + 1G > A, del5395, or 1100delC) (27.3%) and (4.5%), respectively, than in those with *CHEK2* WT (23.8%) and (0.8%), respectively. Moreover, aggressive forms of PTC were more common in those with a heterozygous *CHEK2* truncating mutation than in those with *CHEK2* WT (2.3% versus 0.7%, respectively). Vascular invasion was more common in those with a heterozygous truncating mutation (IVS2 + 1G > A, del5395, or 1100delC) than in those with *CHEK2* WT (*p* = 0.0002). The heterozygous truncating *CHEK2* mutation (IVS2 + 1G > A, del5395, or 1100delC) was significantly associated with an advanced (IR or HR) initial risk of stratification (*p* = 0.0207). There was no significant relationship between 131-I treatment or response to primary treatment (excellent, indeterminate, biochemically incomplete, or structurally incomplete) in patients with a heterozygous truncating *CHEK2* mutation (IVS2 + 1G > A, del5395, or 1100delC) in comparison to *CHEK2* WT patients. There were no significant differences in disease outcome at final follow-up between the remission (NED) and no-remission (indeterminate, biochemically incomplete, or structurally incomplete) categories. Recurrence after NED was observed in 1.3% (17 out of 1290) of *CHEK2* WT patients and in none of the patients with a heterozygous *CHEK2* truncating mutation (IVS2 + 1G > A, del5395, or 1100delC) (*p* = 1.0). Death from other causes occurred in 1.1% (15/1292) of *CHEK2* WT patients and in none of the patients with a heterozygous *CHEK2* truncating mutation (IVS2 + 1G > A, del5395, or 1100delC) (*p* = 1.0).

#### 3.1.5. Relationship between the Heterozygous Truncating *CHEK2* Mutation Variants (IVS2 + 1G > A, del5395, and 1100delC) and Heterozygous Missense Mutation I157T with Respect to Clinicopathological Characteristics, Responses to Therapy, and Disease Outcomes

The clinicopathological characteristics, responses to treatment, and disease outcomes of 44 patients with heterozygous truncating *CHEK2* mutation variants (IVS2 + 1G > A, del5395, or 1100delC) were compared with those harboring the heterozygous missense I157T mutation (Table 3). There was no significant relationship between the two groups in terms of sex, age at diagnosis, tumor size, multifocality, LN metastases, distant metastases, extrathyroidal extension, and more advanced clinical stage. Differences in histological variant of PTC were statistically significant (*p* = 0.0219). The classic histological variant of PTC was more common in patients with heterozygous missense I157T mutation (74.7%) than in patients with a heterozygous *CHEK2* truncating mutation (IVS2 + 1G > A, del5395, or 1100delC) (61.4%). The follicular and oxyphilic variants were more common in patients with a heterozygous *CHEK2* truncating mutation (IVS2 + 1G > A, del5395, or 1100delC) (27.3%) and (4.5%), respectively, than in those with heterozygous missense mutation I157T (21.4%) and (0%), respectively. Moreover, aggressive forms of PTC were more common in those with a heterozygous *CHEK2* truncating mutation than in those with a heterozygous missense I157T mutation (2.3% versus 0.5%, respectively). Vascular invasion was more common in those with a heterozygous truncating mutation (IVS2 + 1G > A, del5395, or 1100delC) than in those with a heterozygous missense I157T mutation (*p* = 0.0004). The heterozygous truncating *CHEK2* mutation (IVS2 + 1G > A, del5395, or 1100delC) was not associated with an advanced (IR or HR) initial risk of stratification (*p* = 0.0567) or 131-I treatment. There was a significant relationship between the response to primary treatment (excellent, indeterminate, biochemically incomplete, or structurally incomplete) and heterozygous truncating *CHEK2* mutation (*p* = 0.0225). There were no significant differences in disease outcome at final follow-up between the remission (NED) and no remission (indeterminate, biochemically incomplete, or structurally incomplete) categories. Recurrence after NED was observed in 1.1% (2 out of 182) of heterozygous missense I157T mutation patients and in none of the patients with a heterozygous *CHEK2* truncating mutation (IVS2 + 1G > A, del5395, or 1100delC) (*p* = 1.0). Death from other causes occurred in 0.5% (1/182) of heterozygous missense I157T mutation patients and in none of the patients with a heterozygous *CHEK2* truncating mutation (IVS2 + 1G > A, del5395, or 1100delC) (*p* = 1.0).

#### 3.1.6. The Impact of *CHEK2* Mutation Status on Vascular Invasion, High and Intermediate Risk of Recurrence/Persistence According to ATA, response to Initial Therapy and Disease Outcome in Univariate and Multivariate Regression Analysis

Heterozygous truncating *CHEK2* mutation variants (IVS2 + 1G > A, del5395, and 1100delC) had a significant impact on vascular invasion in univariate analysis (OR, 5.64; 95% CI, 2.59–12.29; *p* < 0.0001) and in multivariate analysis (OR, 6.91; 95% CI, 2.81–17.03; *p* < 0.0001), whereas the I157T missense mutation had no effect on vascular invasion in univariate and multivariate analysis (Table 4). The heterozygous truncating mutation and the missense I157T mutation and had no effect on the response to the initial therapy and the outcome of the disease (Table 5 and Table 6). Moreover, heterozygous truncating mutation was a significant predictor of intermediate or high risk of recurrence/persistence, when evaluated according to the ATA risk stratification system (Table S1) (OR, 2.01; 95% CI, 1.1–3.68; *p* = 0.0231) in univariate analysis and in multivariate analysis (OR, 1.92; 95% CI, 1.01–3.67; *p* = 0.0481), whereas the missense mutation was not a significant predictor of recurrence/persistence risk according to ATA in univariate and multivariate analysis (Table S1).

#### 3.1.7. Clinical Characteristics of PTC Patients Carrying Two *CHEK2* Mutations

Fourteen out of 1547 (0.9%) patients in the study group carried two *CHEK2* mutations (five women and two men had a homozygous *CHEK2* I157T mutation, and two men were carriers of both IVS2 + 1G and del5395). Three women carried both IVS2 + 1G and I157T, and three women carried both del5395 and I157T. The clinical characteristics of patients carrying two *CHEK2* mutations are presented in Table 7. There was no significant relationship between homozygotes and patients carrying two different mutations in terms of clinical and pathological characteristics, response to primary treatment, or disease outcome (persistent/recurrent disease/death).

## 4. Discussion

DNA repair is a fundamental process that protects genes from becoming unstable. Disrupting the DNA repair system, which includes the *CHEK2* gene, leads to genomic instability, which is responsible for tumor progression and transformation of normal cells into cancer cells [51,52]. Polymorphisms and mutations in the *CHEK2* gene can lead to occurrence of sporadic cancers; however, they also cause predisposition to familial types of cancer, including thyroid cancer [32,37,39]. Here, we found that the overall frequency of mutations in the *CHEK2* gene in PTC patients was 15.5%, which is in agreement with previous study results [23,43]. However, the TCGA 2014 study conducted in a North American population found that the *CHEK2* mutation was present in only 1.2% of PTC patients, whereas Alzahrani et al. and Fayaz et al. found no mutations in the *CHEK2* gene in Middle Eastern populations [38,41,42]. This may be related to geographic or ethnic differences, or (albeit rather less likely) to different techniques used to detect mutant alleles. In our study, as well as in our previous studies, we used Sanger sequencing rather than Next Generation Sequencing (NGS); this is because the sensitivity of Sanger sequencing is 20% of the allele frequency, making it suitable for detecting a germline mutation of around 50% of the allele frequency [23,43,53]. This was confirmed in the TCGA 2014 study in which mutations detected by NGS were confirmed by Sanger sequencing and, in some cases in which clonality was investigated, the clonal fraction was high (70–100%) [42]. Alzahrani et al. (Saudi Arabia) used a technique similar to our own, whereas Fayaz et al. (Iran) used the PCR–high-resolution melting (HRM) technique [38,41]. Neither of these studies detected mutations in the *CHEK2* gene. In our study, we found that patients harboring different mutations in the *CHEK2* gene were at different levels of risk of developing PTC. *CHEK2* truncating mutations (1100delC, IVS2 + 1G> A, and del5395) were associated with a higher risk of PTC (OR, 4.54; *p* = 0.0116), whereas the missense I157T mutation was associated with a lower risk (OR, 2.47; *p* = <0.0001). This is comparable to the results of our previous study that examined fewer PTC patients [23]. In another study, Kaczmarek-Ryś et al., examined 602 patients with differentiated thyroid cancer (DTC) and 829 healthy controls [39]. They found that the heterozygous c470C (I157T) variant increases the risk of DTC by almost 2-fold (OR, 1.81; *p* = 0.004), while the homozygous c470C (I157T) variant, observed in three women with DTC (0.57%), increased the risk by approximately 13 times (OR, 12.8; *p* = 0.019); there was no association in men [39]. Our results confirm those published by Wójcicka et al., who examined a large group of 1781 patients with PTC and 2081 healthy people. They showed that the rs17879961 (I157T) variant of the *CHEK2* gene is a factor predisposing to PTC (OR, 2.2; *p* = 2.37 × 10^−10^) [22]. However, truncating mutations (1100delC, IVS2 + 1G > A, and del5395) in *CHEK2* were not analyzed in those studies. To the best of our knowledge, our study is the first to analyze both missense I157T and truncating mutations (1100delC, IVS2 + 1G > A, and del5395) in the *CHEK2* gene in a large number of PTC patients. Previously, we found mutations in 65/427 PTC patients [43].

Due to the small number of patients with truncating mutations (1100delC, IVS2 + 1G > A, and del5395) in the *CHEK2* gene, we included all patients with both missense I157T and truncating *CHEK2* mutations in the analysis. A few studies have analyzed the effect of mutations in the *CHEK2* gene on the clinicopathological features and course of PTC, but no studies have analyzed these two variants separately due to the fact that truncating mutations are less common than the I157T missense mutation. Here, we did not find a relationship between mutations in the *CHEK2* gene, analyzing missense I157T + truncating mutations (1100delC, IVS2 + 1G > A, and del5395), with aggressive clinicopathological features and disease course, which is the same result as that reported in our previous work [43]. We also identified an association between the missense I157T *CHEK2* heterozygous mutation and aggressive clinicopathological features, a finding similar to that reported by Kaczmarek-Ryś et al. [39]. In that study, the authors found a relationship between the c.407C allele (I157T) and age of onset [39]. The c.407C (I157T) allele was more common in DTCs in patients aged 51–60 years (*p* = 0.016). However, neither we nor Wójcicka et al. found such a relationship [22]. In our previous study, we found that the *CHEK2* gene missense I157T plus truncating mutations (1100delC, IVS2 + 1G > A, and del5395) were more common in those with the classic PTC variant than in those with other PTC variants [43]. Again, no such relationship was found in the present study.

In addition, we found no relationship when analyzing the missense variant of the I157T *CHEK2* heterozygote separately. It is worth noting that the TCGA 2014 study suggested that the *CHEK2* pathway may be associated specifically with the follicular variant of PTC [42]. Our own results showed that truncating mutations 1100delC, IVS2 + 1G > A, and del5395 in the *CHEK2* gene were more common in the follicular, oxyphilic, and aggressive variants than in the classic PTC variant. Although the univariate and multivariate analyses showed a noticeable relationship between the truncating mutations and vascular invasion, initial risk stratification (intermediate- or high-risk), there was no significant differences in response to primary treatment and outcome of the disease. It is likely that reports of an association between vascular invasion and a more aggressive PTC phenotype were influenced by inclusion of this histopathological factor as an obligatory element in the ATA risk stratification guidelines [12]. In addition to the impact of vascular invasion on an initial prognosis of PTC, there are indications that responses to primary treatment, which modify the initial risk and change the patient’s prognosis, may also depend on vascular invasion. Indeed, Gardner et al., Falvo et al. and Mete et al. (but not Furlan et al. and Akslen et al.) reported that vascular invasion is associated with an unfavorable clinical course (i.e., a worse outcome of the disease) [54,55,56,57,58]. In our study, vascular invasion (considered as an adverse prognostic factor), found more often in patients with truncating mutation, did not lead to worse response to initial therapy and did not influence outcome of the disease (remission vs. no remission). This is in line with Tuttle et al., Momesso et al., Castagna et al. and Kowalska et al., suggesting that the delayed risk stratification system takes into account the primary treatment response and correlates much better with the disease outcome (remission vs. no remission) than the system of initial risk stratification by ATA [47,50,59,60].

In our study, we found that 14/1547 (0.9%) patients had two mutations in the *CHEK2* gene. We identified seven patients with the homozygous I157T mutation, five with both the I157T missense mutation and one of the truncating mutations (1100delC, IVS2 + 1G > A, and del5395), and two with two different truncating mutations in the *CHEK2* gene. Due to the small number of patients with two mutations (14/1547), we did not include them in the comparisons with patients with *CHEK2* WT. It is impossible to draw a definitive conclusion regarding the effect of homozygous status and coexistence of both mutations on disease course based on just seven cases from each group. The authors of other studies did not include patients with the homozygous I157T mutation in their detailed analysis due to the small numbers involved [23,39,43]. Kaczmarek-Ryś et al. showed that patients carrying the homozygous variant were, on average, 7 years younger than other patients participating in the study [39]. They concluded that being homozygous could predispose to DTC at a younger age. However, we and others observed no such relationship [23,43].

The present study has several strengths. First, it includes a large (1547) and homogeneous ethnic group of Caucasian patients from a single center in Poland, all of whom were diagnosed and then treated in accordance with current guidelines for thyroid cancer. Second, there are no studies in the literature that report such a large group of patients harboring a mutation in the *CHEK2* gene (240/1547) (either the I157T missense mutation or truncating mutations 1100delC, IVS2 + 1G > A, and del5395). Third, the results of studies of pathogenic mutations in the *CHEK2* gene were obtained using the same method used herein, suggesting that methodology has no bearing on the final result.

This work also has some limitations. We mainly included those with low-risk tumors (68.2%) and a large number of microcarcinomas (≤1 cm, 63.8%). This may have an impact on the results. However, a large number of new PTC microcarcinomas are observed worldwide, mainly due to overdiagnosis [2,6,61,62]. Another limitation may be the relatively small number of cases with a truncating mutation (1100delC, IVS2 + 1G > A, and del5395). Due to limited financial resources (this study did not receive a specific grant from any funding agency), we were unable to test expression of *CHEK2* and p53 proteins in PTC tumors by immunohistochemistry, which could be an attractive strategy for improving risk stratification in patients. It will be interesting to evaluate the expression of *CHEK2* and p53 proteins in PTC tumors in future studies.

Nevertheless, we believe that the data presented herein are interesting because they come from one center and the follow-up period is long (6 years; range 1–34). Thus, these data are suitable for inclusion in future meta-analyses.

To verify the effect of the truncating mutation on clinical course and outcome in PTC patients, it is necessary to conduct studies on a larger number of PTC patients. It is also important to determine whether germline mutations, in particular truncating mutations in the *CHEK2* gene, could be used to predict the onset and development of PTC in healthy individuals. It would be helpful if these mutations are detected prior to surgery because the presence of a high-risk lesion is very likely. Genotyping of larger cohorts is needed to examine tumor characteristics in carriers of these mutations.

## 5. Conclusions

Taken together, the results indicate that *CHEK2* truncating mutations are associated with a higher risk of PTC development than the *CHEK2* I157T missense mutation. The truncating mutations are strongly associated with vascular invasion, a factor related to poor prognosis, in comparison to the missense mutation. The truncating mutations are more often associated with intermediate and high risk of recurrence/persistence according to the ATA initial risk stratification system. Both the *CHEK2* truncating and the *CHEK2* I157T missense mutation had no effect on the poorer primary treatment response and outcome of the disease.

## Figures and Tables

**Table 1:** Characteristics of the 1547 patients with papillary thyroid carcinoma (PTC).

Characteristic	Total (*n* = 1547)
Sex	
Female	1358 (87.8%)
Male	189 (12.2%)
Age at diagnosis (years)	
Mean (SD)	48.7 (13.6)
Median (Q1–Q3)	50.0 (39.0–59.0)
Range	15.0–85.0
Age	
<55	990 (64.0%)
≥55	557 (36.0%)
Tumor diameter (mm)	
Mean (SD)	11.6 (11.5)
Median (Q1–Q3)	8.0 (5.0–14.0)
Range	0.3–110.0
Tumor diameter (mm)	
≤10	987 (63.8%)
>10–20	352 (22.8%)
>20–40	162 (10.5%)
>40	46 (3.0%)
Papillary cancer histologic variant	
Classic	1111 (71.8%)
Follicular	363 (23.5%)
Oxyphilic	13 (0.8%)
Diffuse sclerosing	9 (0.6%)
Tall cell	4 (0.3%)
Other *	47 (3.0%)
Multifocality	
No	1143 (73.9%)
Yes	404 (26.1%)
Nodal metastases **	
N0a	801 (51.8%)
N0b	521 (33.7%)
N1a	124 (8.0%)
N1b	101 (6.5%)
Distant metastases	
No	1532 (99.0%)
Yes	15 (1.0%)
Extrathyroidal extension	
Negative	1250 (80.8%)
Microscopic	272 (17.6%)
Gross	25 (1.6%)
Vascular invasion	
No	1475 (95.3%)
Yes	72 (4.7%)
Tumor stage	
pT1a	980 (63.3%)
pT1b	349 (22.6%)
pT2	152 (9.8%)
pT3a	42 (2.7%)
pT3b	17 (1.1%)
pT4a	6 (0.4%)
pT4b	1 (0.1%)
TNM (8th edition)	
I	1466 (94.8%)
II	71 (4.6%)
III	2 (0.1%)
IVa	1 (0.1%)
IVb	7 (0.5%)
*CHEK2* mutation status	
*CHEK2* WT	1307 (84.5%)
*CHEK2* mutation (any)	240 (15.5%)
*CHEK2* I157T missense mutation (any)	189 (12.3%)
I157T heterozygous	182 (11.8%)
I157T homozygous	7 (0.5%)
*CHEK2* truncating heterozygous mutation (any)	44 (2.8%)
IVS2 + 1G > A	18 (1.2%)
Del5395	10 (0.6%)
1100delC	16 (1.0%)
Coexistence of two heterozygous *CHEK2* mutations	7 (0.5%)
I157T and IVS2 + 1G > A	3 (0.2%)
I157T and Del5395	2 (0.1%)
IVS2 + 1G > A and Del5395	2 (0.1%)
ATA initial risk stratification system	
Low	1055 (68.2%)
Intermediate	427 (27.6%)
High	65 (4.2%)
Radioactive iodine therapy (I-131)	
No	493 (31.9%)
Yes	1054 (68.1%)
More than one course of radioactive iodine therapy (I-131)	
No	1371 (88.6%)
Yes	176 (11.4%)
Response to therapy	
Excellent	1259 (81.4%)
Indeterminate	234 (15.1%)
Biochemically incomplete	22 (1.4%)
Structurally incomplete	32 (2.1%)
Final follow-up (31 May 2020)	
NED	1423 (92.0%)
Indeterminate	102 (6.6%)
Biochemically persistent disease	12 (0.8%)
Structurally persistent disease	10 (0.6%)
Follow-up, recurrence after NED	
No	1528 (98.8%)
Yes	19 (1.2%)
Death	
No	1531 (99.0%)
TC (unrelated)	16 (1.0%)
Median follow-up time, years (range)	6.0 (1.0–34.0)

* Warthin-like (*n* = 6); cribriforme morular (*n* = 3); solid (*n* = 2); mixed variant (classic and follicular) (*n* = 36).** N0a, one or more cytologically or histologically confirmed benign lymph nodes; N0b, no radiologic or clinical evidence of locoregional lymph node metastasis; N1a–N1b, metastasis to regional lymph nodes; ATA, American Thyroid Association, determined according to the 8th edition of the American Joint Committee on Cancer/Union for International Cancer Control (tumor-node-metastasis) TNM staging system; *CHEK2* WT (wild-type) = cases without the following mutations: I157T, 1100delC, IVS2 + 1G > A, del5395; SD, standard deviation; NED, no evidence of disease.

**Table 2:** Relationship between *CHEK2* mutation genotype and PTC risk.

*CHEK2* Mutation Status	Control Group (*n* = 468)	Study Group (*n* = 1547)	*p* Value (Chi-Square or Fisher’s Exact Test)	OR	95% CI	*p* Value (Logistic Regression Model)
*CHEK2* mutation (any)	28 (6.0%)	240 (15.5%)	<0.0001	2.89	1.92–4.33	<0.0001
*CHEK2 WT*	440 (94%)	1307 (84.5%)	<0.0001	0.35	0.23–0.52	<0.0001
*CHEK2* Detailed Mutation Status:						
Missense I157T	25 (5.3%)	189 (12.3%)	<0.0001	2.47	1.60–3.79	<0.0001
I157T heterozygous	25 (5.3%)	182 (11.8%)	<0.0001	2.36	1.53–3.63	<0.0001
I157T homozygous	0 (0.0%)	7 (0.5%)	0.3641	Not calculable (0 in cell)		
*CHEK2* Heterozygous Truncating Mutation	3 (0.6%)	44 (2.8%)	0.0057	4.54	1.40–14.68	0.0116
IVS2 + 1G > A	1 (0.2%)	18 (1.2%)	0.0261	7.05	0.95–52.31	0.0562
Del5395	2 (0.4%)	10 (0.6%)	0.3888	2.13	0.48–9.4	0.319
1100delC	0 (0.0%)	16 (1.0%)	0.0315	Not calculable (0 in cell)		
*CHEK2* Missense I157T + Truncating Mutations	0 (0.0%)	5 (0.3%)	0.5961	Not calculable (0 in cell)		
IVS2 + 1G > A and I157T	0 (0.0%)	3 (0.2%)	1.0	Not calculable (0 in cell)		
del5395 and I157T	0 (0.0%)	2 (0.1%)	1.0	Not calculable (0 in cell)		
Coexistence of Two Truncating Mutations (IVS2 + 1G > A +Del5395)	0 (0.0%)	2 (0.1%)	1.0	Not calculable (0 in cell)		

CI, confidence interval; OR, odds ratio; *CHEK2* WT (wild-type) = cases without the following mutations: I157T, 1100delC, IVS2 + 1G > A, or del5395.

**Table 3:** Impact of different *CHEK2* variants on clinicopathological features, response to therapy, and disease outcome.

Feature	A *CHEK2* WT (*n* = 1307)	B ANY *CHEK2* Mutation (*n* = 240)	C Missense *CHEK2* I157T Heterozygous (*n* = 182)	D Heterozygous Truncating *CHEK2* Mutation IVS2 + 1G > A, Del5395, 1100delC) (*n* = 44)	*p*-Value			
					A vs. B	A vs. C	A vs. D	C vs. D
Sex					0.1164	0.1869	0.0378	0.2101
Female	1140 (87.2%)	218 (90.8%)	165 (90.7%)	43 (97.7%)				
Male	167 (12.8%)	22 (9.2%)	17 (9.3%)	1 (2.3%)				
Age at diagnosis (years)					0.2443	0.4755	0.2569	0.4662
Mean (SD)	48.6 (13.7)	49.5 (12.8)	49.3 (12.7)	50.8 (12.1)				
Median (Q1–Q3)	50.0 (39.0, 58.0)	51.0 (41.0, 59.0)	50.5 (40.2, 58.8)	50.5 (43.5, 61.0)				
Range	15.0–85.0	18.0–76.0	18.0–76.0	23.0–70.0				
Age					0.2670	0.6091	0.2907	0.4765
<55	844 (64.6%)	146 (60.8%)	114 (62.6%)	25 (56.8%)				
≥55	463 (35.4%)	94 (39.2%)	68 (37.4%)	19 (43.2%)				
Tumor diameter (mm)					0.9617	0.8702	0.9973	0.8440
Mean (SD)	11.5 (11.1)	12.4 (13.4)	12.1 (13.4)	13.6 (14.7)				
Median (Q1–Q3)	8.0 (5.0, 14.0)	8.0 (5.0, 15.0)	9.0 (4.0, 15.0)	7.0 (5.0, 20.2)				
Range	0.3–110.0	0.3–84.0	0.3–84.0	1.0–80.0				
Tumor diameter (mm)					0.5255	0.6763	0.0356	0.0634
≤10	833 (63.7%)	154 (64.2%)	117 (64.3%)	28 (63.6%)				
>10–20	304 (23.3%)	48 (20.0%)	40 (22.0%)	5 (11.4%)				
>20–40	133 (10.2%)	29 (12.1%)	17 (9.3%)	10 (22.7%)				
>40 mm	37 (2.8%)	9 (3.8%)	8 (4.4%)	1 (2.3%)				
Papillary cancer histology variant					0.8195	0.836	0.0397	0.0219
Classic	936 (71.6%)	175 (72.9%)	136 (74.7%)	27 (61.4%)				
Follicular	311 (23.8%)	52 (21.7%)	39 (21.4%)	12 (27.3%)				
Oxyphilic	11 (0.8%)	2 (0.8%)	0 (0.0%)	2 (4.5%)				
Diffuse sclerosing	7 (0.5%)	2 (0.8%)	1 (0.5%)	0 (0.0%)				
Tall cell	3 (0.2%)	1 (0.4%)	0 (0.0%)	1 (2.3%)				
Other *	39 (3.0%)	8 (3.3%)	6 (3.3%)	2 (4.5%)				
Multifocality					0.4894	0.7426	0.5754	0.7264
No	970 (74.2%)	173 (72.1%)	133 (73.1%)	31 (70.5%)				
Yes	337 (25.8%)	67 (27.9%)	49 (26.9%)	13 (29.5%)				
Nodal metastases **					0.7774	0.8779	0.4645	0.7084
N0a	681 (52.1%)	120 (50.0%)	92 (50.5%)	19 (43.2%)				
N0b	439 (33.6%)	82 (34.2%)	62 (34.1%)	16 (36.4%)				
N1a	101 (7.7%)	23 (9.6%)	17 (9.3%)	5 (11.4%)				
N1b	86 (6.6%)	15 (6.2%)	11 (6.0%)	4 (9.1%)				
Distant metastases					0.1475	0.2402	1	NA
No	1292 (98.9%)	240 (100.0%)	182 (100.0%)	44 (100.0%)				
Yes	15 (1.1%)	0 (0.0%)	0 (0.0%)	0 (0.0%)				
Extrathyroidal extension					0.6242	0.8426	0.1330	0.1736
Negative	1061 (81.2%)	189 (78.8%)	146 (80.2%)	32 (72.7%)				
Microscopic	225 (17.2%)	47 (19.6%)	34 (18.7%)	10 (22.7%)				
Gross	21 (1.6%)	4 (1.7%)	2 (1.1%)	2 (4.5%)				
Vascular invasion					0.2017	0.5039	0.0002	0.0004
No	1250 (95.6%)	225 (93.8%)	176 (96.7%)	35 (79.5%)				
Yes	57 (4.4%)	15 (6.2%)	6 (3.3%)	9 (20.5%)				
Tumor stage					0.7318	0.9086	0.0728	0.0850
pT1a	827 (63.3%)	153 (63.8%)	116 (63.7%)	28 (63.6%)				
pT1b	300 (23.0%)	49 (20.4%)	41 (22.5%)	5 (11.4%)				
pT2	126 (9.6%)	26 (10.8%)	16 (8.8%)	8 (18.2%)				
pT3a	34 (2.6%)	8 (3.3%)	7 (3.8%)	1 (2.3%)				
pT3b	13 (1.0%)	4 (1.7%)	2 (1.1%)	2 (4.5%)				
pT4a	6 (0.5%)	0 (0.0%)	0 (0.0%)	0 (0.0%)				
pT4b	1 (0.1%)	0 (0.0%)	0 (0.0%)	0 (0.0%)				
TNM (8th edition)					0.8089	1	0.6076	0.4514
I	1238 (94.7%)	228 (95.0%)	174 (95.6%)	41 (93.2%)				
II	59 (4.5%)	12 (5.0%)	8 (4.4%)	3 (6.8%)				
III	2 (0.2%)	0 (0.0%)	0 (0.0%)	0 (0.0%)				
IVa	1 (0.1%)	0 (0.0%)	0 (0.0%)	0 (0.0%)				
IVb	7 (0.5%)	0 (0.0%)	0 (0.0%)	0 (0.0%)				
ATA initial risk stratification system					0.24710	0.74383	0.0207	0.0567
Low	899 (68.8%)	156 (65.0%)	123 (67.6%)	23 (52.3%)				
Intermediate + High	408 (31.2%)	84 (35.0%)	59 (32.4%)	21 (47.7%)				
Radioactive iodine therapy (I-131)					0.4057	0.2474	0.7893	0.4399
No	411 (31.4%)	82 (34.2%)	65 (35.7%)	13 (29.5%)				
Yes	896 (68.6%)	158 (65.8%)	117 (64.3%)	31 (70.5%)				
More than on course of I-131					0.1632	0.1505	0.7204	0.2581
No	1152 (88.1%)	219 (91.2%)	167 (91.8%)	38 (86.4%)				
Yes	155 (11.9%)	21 (8.8%)	15 (8.2%)	6 (13.6%)				
Response to therapy					0.7652	0.2369	0.1903	0.0225
Excellent	1060 (81.1%)	199 (82.9%)	156 (85.7%)	32 (72.7%)				
Indeterminate	198 (15.1%)	36 (15.0%)	24 (13.2%)	9 (20.5%)				
Biochemically incomplete	20 (1.5%)	2 (0.8%)	0 (0.0%)	2 (4.5%)				
Structurally incomplete	29 (2.2%)	3 (1.2%)	2 (1.1%)	1 (2.3%)				
Final follow-up (31 May 2020)					0.1122	0.2731	0.4120	0.1553
Remission (NED)	1198 (91.7%)	225 (93.8%)	173 (95.1%)	39 (88.6%)				
No remission ***	109 (8.3%)	15 (6.2%)	9 (4.9%)	5 (11.4%)				
Follow-up, recurrence					0.7547	1	1	1
No	1290 (98.7%)	238 (99.2%)	180 (98.9%)	44 (100.0%)				
Yes	17 (1.3%)	2 (0.8%)	2 (1.1%)	0 (0.0%)				
Death					0.4914	0.7094	1	1
No	1292 (98.9%)	239 (99.6%)	181 (99.5%)	44 (100.0%)				
TC-unrelated	15 (1.1%)	1 (0.4%)	1 (0.5%)	0 (0.0%)				
Follow-up (years)					0.9494	0.8180	0.7305	0.6735
Mean (SD)	7.5 (5.4)	7.7 (5.9)	7.7 (5.8)	7.9 (6.9)				
Median (Q1, Q3)	6.0 (3.0, 11.0)	6.0 (3.0, 12.0)	6.0 (3.0, 12.0)	5.0 (2.8, 12.0)				
Range	1.0–32.0	1.0–34.0	1.0–34.0	1.0–25.0				

* Warthin-like (*n* = 6); cribriforme morular (*n* = 3); solid (*n* = 2); mixed variants (classic and follicular) (*n* = 36). ** N0a, one or more cytologically or histologically confirmed benign lymph nodes; N0b, no radiologic or clinical evidence of locoregional lymph node metastasis; N1a–N1b, metastasis to regional lymph nodes. *** No remission—Indeterminate, Biochemically persistent disease, Structurally persistent disease. ATA, American Thyroid Association, determined according to the 8th edition of the American Joint Committee on Cancer/Union for International Cancer Control TNM staging system; *CHEK2* WT = wild-type, cases without the following mutations: I157T, 1100delC, IVS2 + 1G > A, del5395; SD, standard deviation; NED, no evidence of disease; NA, not available.

**Table 4:** Risk factors for vascular invasion.

Feature	Details	Univariable OR	95% CI	*p*	Multivariable OR	95% CI	*p*
Male gender	no	Ref. level			Ref. level		
	yes	2	1.11–3.61	0.0216	1.23	0.61–2.49	0.5592
Age at diagnosis (years)		0.99	0.97–1.01	0.3112			
Tumor diameter (mm)		1.05	1.03–1.06	<0.0001	1.03	1.01–1.04	0.002
Papillary cancer histologic variant	1. Classic	Ref. level			Ref. level		
	2. Follicular	0.87	0.48–1.57	0.643	1.34	0.69–2.57	0.3876
	3. Oxyphilic	NA (0 in cell)			NA (0 in cell)		
	4. Diffuse sclerosing	6.71	1.32–34.06	0.0216	5.21	0.85–31.74	0.0736
	5. Tall cell	NA (0 in cell)			NA (0 in cell)		
	6. Other	1.37	0.41–4.57	0.6055	1.14	0.3–4.34	0.8462
Extrathyroidal extension	1. Negative	Ref. level			Ref. level		
	2. Micro	5.64	3.4–9.35	<0.0001	3.06	1.73–5.44	0.0001
	3. Gross	15.15	5.9–38.91	<0.0001	3.62	1.2–10.92	0.0222
Multifocality	no	Ref. level			Ref. level		
	yes	1.99	1.22–3.23	0.0055	1.32	0.76–2.3	0.3184
Nodal metastases N1a or N1b	no	Ref. level			Ref. level		
	yes	8.68	5.32–14.16	<0.0001	4.55	2.57–8.05	<0.0001
Distant metastases	no	Ref. level			Ref. level		
	yes	7.75	2.41–24.98	0.0006	3.23	0.79–13.3	0.1042
*CHEK2* mutation status	1. *CHEK2* WT	Ref. level			Ref. level		
	2. heterozygous truncating mutation	5.64	2.59–12.29	<0.0001	6.91	2.81–17.03	<0.0001
	3. Missense *CHEK2* I157T heterozygous	0.75	0.32–1.76	0.5053	0.69	0.28–1.72	0.4263

CI, confidence interval; OR, odds ratio; NA, not available; N1a–N1b, metastasis to regional lymph nodes; heterozygous truncating mutation (IVS2 + 1G > A, Del5395, 1100delC); *CHEK2* WT = wild-type, cases without the following mutations: I157T, 1100delC, IVS2 + 1G > A, del5395.

**Table 5:** Risk factors for non-excellent response (indeterminate, biochemically and structurally incomplete) to initial therapy.

Feature	Details	Univariable OR	95% CI	*p*	Multivariable OR	95% CI	*p*
Male gender	no	Ref. level			Ref. level		
	yes	2.06	1.46–2.91	<0.0001	1.63	1.11–2.39	0.013
Age at diagnosis (years)		0.99	0.98–0.99	0.0021	0.99	0.98–1	0.0156
Tumor diameter (mm)		1.04	1.03–1.05	<0.0001	1.02	1.01–1.03	0.0001
Papillary cancer histologic variant	1. Classic	Ref. level			Ref. level		
	2. Follicular	0.69	0.5–0.96	0.0256	0.84	0.6–1.19	0.3361
	3. Oxyphilic	2.51	0.81–7.75	0.1093	2.41	0.73–7.95	0.148
	4. Diffuse sclerosing	1.34	0.27–6.68	0.7215	0.74	0.13–4.12	0.7304
	5. Tall cell	NA (0 in cell)			NA (0 in cell)		
	6. Other	0.59	0.25–1.4	0.2313	0.45	0.17–1.18	0.105
Extrathyroidal extension	1. Negative	Ref. level			Ref. level		
	2. Microscopic	2.74	2.03–3.7	<0.0001	1.89	1.35–2.64	0.0002
	3. Gross	7.3	3.26–16.32	<0.0001	3.21	1.28–8.09	0.0132
Vascular invasion	no	Ref. level			Ref. level		
	yes	4.06	2.5–6.58	<0.0001	1.68	0.97–2.91	0.0665
Multifocality	no	Ref. level			Ref. level		
	yes	1.89	1.44–2.49	<0.0001	1.64	1.22–2.21	0.0011
Nodal metastases N1a or N1b	no	Ref. level			Ref. level		
	yes	4.13	3.04–5.61	<0.0001	2.23	1.56–3.19	<0.0001
Distant metastases	no	Ref. level					
	yes	NA (0 in cell)					
ATA initial risk (intermediate or high)	no	Ref. level					
	yes	5.03	3.83–6.61	<0.0001			
*CHEK2* mutation status	1. *CHEK2* WT	Ref. level					
	2. heterozygous truncating mutation	1.61	0.82–3.17	0.1688			
	3. Missense *CHEK2* I157T heterozygous	0.72	0.46–1.11	0.1334			

CI, confidence interval; OR, odds ratio; NA, not available; N1a–N1b, metastasis to regional lymph nodes; heterozygous truncating mutation (IVS2 + 1G > A, Del5395, 1100delC); *CHEK2* WT = wild-type, cases without the following mutations: I157T, 1100delC, IVS2 + 1G > A, del5395; ATA, American Thyroid Association, determined according to the 8th edition of the American Joint Committee on Cancer/Union for International Cancer Control TNM staging system.

**Table 6:** Risk factors for patients without remission (indeterminate, biochemically and structurally persistent disease) in final follow-up.

Feature	Details	Univariable OR	95% CI	*p*	Multivariable OR	95% CI	*p*
Male gender	no	Ref. level			Ref. level		
	yes	2.61	1.68–4.07	<0.0001	1.88	1.15–3.08	0.0124
Age at diagnosis (years)		0.99	0.98–1.01	0.2978			
Tumor diameter (mm)		1.04	1.03–1.05	<0.0001	1.02	1–1.03	0.0109
Papillary cancer histologic variant	1. Classic	Ref. level					
	2. Follicular	0.63	0.39–1.02	0.0621			
	3. Oxyphilic	0.85	0.11–6.62	0.8776			
	4. Diffuse sclerosing	1.46	0.18–11.98	0.725			
	5. Tall cell	NA (0 in cell)					
	6. Other	0.45	0.11–1.9	0.2796			
Extrathyroidal extension	1. Negative	Ref. level			Ref. level		
	2. Micro	2.87	1.91–4.32	<0.0001	1.64	1.03–2.6	0.0357
	3. Gross	8.98	3.84–21.02	<0.0001	2.58	0.93–7.16	0.0679
Vascular invasion	no	Ref. level			Ref. level		
	yes	4.68	2.67–8.2	<0.0001	1.53	0.79–2.95	0.2032
Multifocality	no	Ref. level			Ref. level		
	yes	1.72	1.17–2.53	0.0059	1.25	0.82–1.92	0.2952
Nodal metastases N1a or N1b	no	Ref. level			Ref. level		
	yes	6.18	4.18–9.12	<0.0001	3.61	2.31–5.65	<0.0001
Distant metastases	no	Ref. level			Ref. level		
	yes	7.98	2.79–22.81	0.0001	3.14	0.92–10.71	0.0678
ATA initial risk (intermediate or high)	no	Ref. level					
	yes	5.83	3.9–8.71	<0.0001			
*CHEK2* mutation status	1. *CHEK2* WT	Ref. level					
	2. heterozygous truncating mutation	1.41	0.54–3.65	0.4799			
	3. Missense *CHEK2* I157T heterozygous	0.57	0.28–1.15	0.1166			

CI, confidence interval; OR, odds ratio; NA, not available; N1a–N1b, metastasis to regional lymph nodes; heterozygous truncating mutation (IVS2 + 1G > A, Del5395, 1100delC); *CHEK2* WT = wild-type, cases without the following mutations: I157T, 1100delC, IVS2 + 1G > A, del5395; ATA, American Thyroid Association, determined according to the 8th edition of the American Joint Committee on Cancer/Union for International Cancer Control TNM staging system.

**Table 7:** Clinical characteristics of patients with PTC carrying two *CHEK2* mutations.

Characteristic	I157T Missense *CHEK2* Mutation (Homozygous Variants)(*n* = 7)	Coexistence of Two *CHEK2* Mutations IVS2 + 1G and Del5395 (*n* = 2), IVS2 + 1G > A and I157T (*n* = 3), Del5395 and I157T (*n* = 2) *	*p*-Value
Sex			1
Female	5 (71.4%)	5 (71.4%)	
Male	2 (28.6%)	2 (28.6%)	
Age at diagnosis (years)			0.7494
Mean (SD)	48.6 (15.3)	50.0 (19.4)	
Median (Q1–Q3)	51.0 (43.0, 58.0)	56.0 (37.5, 64.5)	
Range	21.0–66.0	19.0–71.0	
Age (years)			1
<55	4 (57.1%)	3 (42.9%)	
≥55	3 (42.9%)	4 (57.1%)	
Tumor diameter (mm)			0.4382
Mean (SD)	14.3 (11.7)	9.2 (5.3)	
Median (Q1–Q3)	10.0 (7.0, 20.5)	6.0 (6.0, 11.2)	
Range	3.0–32.0	5.0–19.0	
Tumor diameter groups			0.5594
≤10 mm	4 (57.1%)	5 (71.4%)	
>10–20 mm	1 (14.3%)	2 (28.6%)	
>20–40 mm	2 (28.6%)	0 (0.0%)	
Papillary cancer histologic variant			0.4615
Classic	5 (71.4%)	7 (100.0%)	
Follicular	1 (14.3%)	0 (0.0%)	
Diffuse sclerosing	1 (14.3%)	0 (0.0%)	
Multifocality			1
No	5 (71.4%)	4 (57.1%)	
Yes	2 (28.6%)	3 (42.9%)	
Nodal metastases			0.5594
N0a	5 (71.4%)	4 (57.1%)	
N0b	1 (14.3%)	3 (42.9%)	
N1a	1 (14.3%)	0 (0.0%)	
Distant metastases			
No	7 (100.0%)	7 (100.0%)	
Extrathyroidal extension			1
Negative	5 (71.4%)	6 (85.7%)	
Microscopic	2 (28.6%)	1 (14.3%)	
Vascular invasion			
No	7 (100.0%)	7 (100.0%)	
Tumor stage			0.5594
pT1a	4 (57.1%)	5 (71.4%)	
pT1b	1 (14.3%)	2 (28.6%)	
pT2	2 (28.6%)	0 (0.0%)	
TNM (8th edition)			1
I	6 (85.7%)	7 (100.0%)	
II	1 (14.3%)	0 (0.0%)	
ATA			0.5594
Low	4 (57.1%)	6 (85.7%)	
Intermediate	3 (42.9%)	1 (14.3%)	
Radioactive iodine therapy (I-131)			1
No	2 (28.6%)	2 (28.6%)	
Yes	5 (71.4%)	5 (71.4%)	
More than one course of I-131			
No	7 (100.0%)	7 (100.0%)	
Response to therapy			1
Excellent	6 (85.7%)	5 (71.4%)	
Indeterminate	1 (14.3%)	2 (28.6%)	
Final follow-up 31 May 2020			1
NED	6 (85.7%)	7 (100.0%)	
Indeterminate	1 (14.3%)	0 (0.0%)	
Follow-up, recurrence			
No	7 (100.0%)	7 (100.0%)	
Death			
No	7 (100.0%)	7 (100.0%)	
Follow-up (years)			0.3358
Mean (SD)	6.1 (5.3)	8.1 (3.6)	
Median (Q1, Q3)	3.0 (2.0, 10.5)	6.0 (5.5, 11.0)	
Range	1.0–14.0	5.0–13.0	

* Truncating IVS2 + 1G and truncating Del5395 (*n* = 2); Truncating IVS2 + 1G > A and missense I157T heterozygous (*n* = 3); Truncating Del5395 and missense I157T heterozygous (*n* = 2); N0a, one or more cytologically or histologically confirmed benign lymph nodes; N0b, no radiologic or clinical evidence of locoregional lymph node metastasis; N1a–N1b, metastasis to regional lymph nodes; ATA, American Thyroid Association, determined according to the 8th edition of the American Joint Committee on Cancer/Union for International Cancer Control TNM staging system; SD, standard deviation; NED, no evidence of disease.

## Data Availability

Data is contained within the article or supplementary material.

## Supplementary Materials

The following are available online at https://www.mdpi.com/2072-6694/13/3/470/s1, Table S1. Risk factors according to 2015 ATA Initial Risk Stratification System—Intermediate or High Risk.
