# Supplementary material for: Incidence of the CHEK2 Germline Mutation and Its Impact on Clinicopathological Features, Treatment Responses, and Disease Course in Patients with Papillary Thyroid Carcinoma
Source: Cancers (Basel). 2021 Jan 26;13(3):470. doi: 10.3390/cancers13030470 (PMC7865996; doi:10.3390/cancers13030470)
Supplement: Supplementary file 1 [file cancers-13-00470-s001.pdf]

cancers

MDPI

Supplementary Materials: Incidence of the *CHEK2* Germline Mutation and Its Impact on Clinicopathological Features, Treatment Responses, and Disease Course in Patients with Papillary Thyroid Carcinoma

Danuta Gąsior-Perczak, Artur Kowalik, Krzysztof Gruszczyński, Agnieszka Walczyk, Monika Siołek, Iwona Pałyga, Sławomir Trepka, Estera Mikina, Tomasz Trybek, Janusz Kopczyński, Agnieszka Suligowska, Rafał Ślusarczyk, Agnieszka Gonet, Jarosław Jaskulski, Paweł Orłowski, Magdalena Chrapek, Stanisław Góźdź and Aldona Kowalska

Table S1. Risk factors according to 2015 ATA Initial Risk Stratification System—Intermediate or High Risk.

Feature	Details	Univariable OR	95% CI	*p*	Multivariable OR	95% CI	*p*
Male gender	no	Ref. level			Ref. level		
	yes	1.5	1.1–2.06	0.0114	1.2	0.84–1.7	0.3193
Age at diagnosis (years)		0.99	0.99–1	0.2059			
Tumor diameter (mm)		1.07	1.05–1.08	< 0.0001	1.06	1.05–1.08	< 0.0001
Multifocality	no	Ref. level			Ref. level		
	yes	2.05	1.62–2.6	< 0.0001	1.89	1.47–2.42	< 0.0001
CHEK2 mutation status	1. *CHEK2* WT	Ref. level			Ref. level		
	2. heterozygous truncating mutation	2.01	1.1–3.68	0.0231	1.92	1.01–3.67	0.0481
	3. Missense *CHEK2* I157T heterozygous	1.06	0.76–1.47	0.7435	1.04	0.72–1.48	0.8484

CI, confidence interval; OR, odds ratio; NA, not available; N1a–N1b, metastasis to regional lymph nodes; heterozygous truncating mutation (IVS2+1G>A, Del5395, 1100delC); *CHEK2* WT = wild-type, cases without the following mutations: I157T, 1100delC, IVS2+1G > A, del5395; ATA, American Thyroid Association, determined according to the 8th edition of the American Joint Committee on Cancer/Union for International Cancer Control TNM staging system.
